# Robot-assisted endoscopic closure of a antral defect using a single-arm transluminal endoscopic robot

**DOI:** 10.1055/a-2777-4660

**Published:** 2026-02-17

**Authors:** Suhuan Liao, Zichuang Hao, Erzhen Zhong, Miao He, Longbin Huang, Qiuping Qiu, Silin Huang

**Affiliations:** 1 Department of Gastroenterology, South China Hospital, Medical School, Shenzhen University, Shenzhen, China

Endoscopic submucosal dissection (ESD) is the standard technique for resection of gastric superficial tumors. However, closing large post-ESD mucosal defects remains technically challenging because edge inversion and inadequate counter-traction can hinder clip apposition. To address the problem of edge inversion, we used a single-arm transluminal endoscopic robot (EndoFaster, Robo Medical Technology Co, Ltd, Shenzhen, China; Video 1 ), which is externally controlled to manipulate a grasping forceps and deliver precise, multi-directional pulling to achieve reliable defect closure 1 .

Single-arm transluminal endoscopic robot-assisted closure of a post-ESD antral defect. ESD, endoscopic submucosal dissection.Video 1

**Fig. 1:**
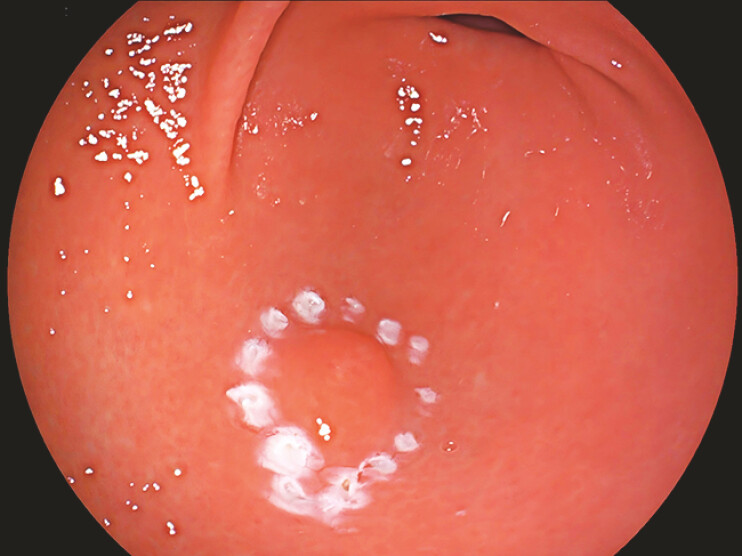
A 12-mm subepithelial lesion in the gastric antrum.

**Fig. 2:**
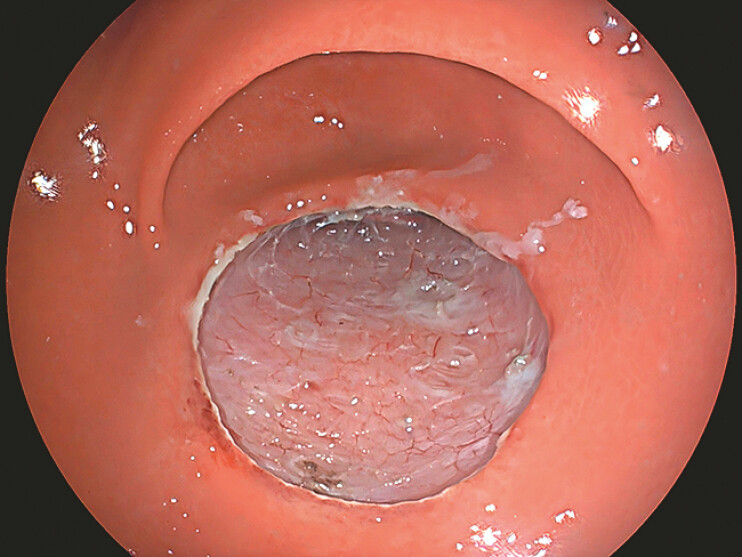
The post-ESD oval defect measured approximately 3 cm × 4 cm (~9.4 cm ^2^ ). ESD, endoscopic submucosal dissection.

**Fig. 3:**
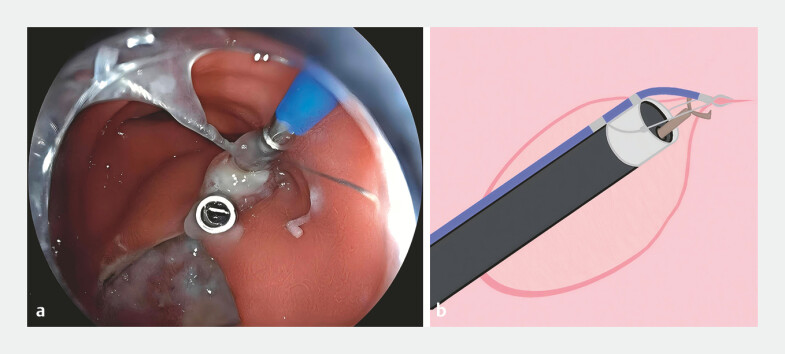
**a, b** The robotic grasping forceps were placed at the 2-o’clock position to pull the distal edge, correcting edge inversion and facilitating deployment of the first through-the-scope.

**Fig. 4:**
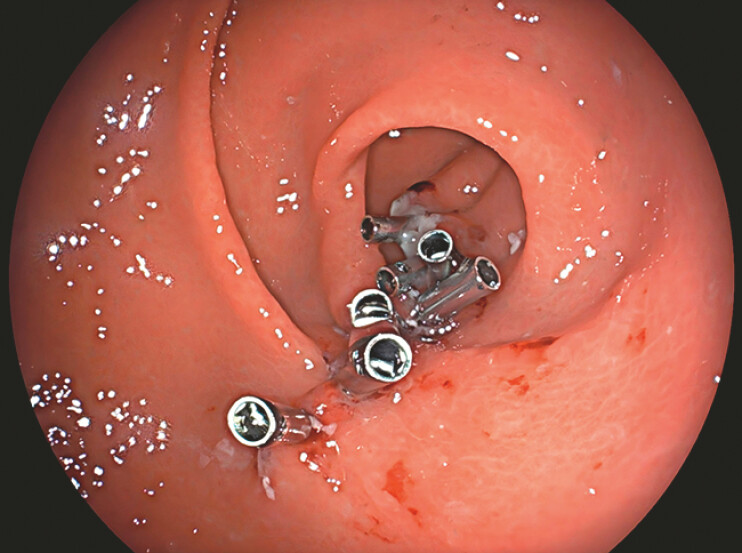
The post-closure wound.

A 25-year-old man had a 12-mm subepithelial lesion in the gastric antrum that was resected en bloc by ESD ( Fig. 1 ). The post-ESD oval defect measured approximately 3 cm × 4 cm (~9.4 cm ^2^ ; Fig. 2 ). To achieve closure for the reduction of postoperative discomfort, a single-arm transluminal endoscopic robot was mounted to the tip of a gastroscope via a soft hood. Under an external joystick control, the robotic grasping forceps were placed at the 1-o’clock position to pull the distal edge, correcting edge inversion and facilitating deployment of the first through-the-scope (TTS) clip ( Fig. 3 ). The grasper was then repositioned to lift the proximal edge on the same side to achieve precise tissue apposition. This traction–clip sequence was repeated circumferentially until complete defect closure was achieved within 5 minutes, using 8 TTS clips ( Fig. 4 ).

Robot-assisted traction markedly improved the visualization and alignment of the mucosal edges, enabling secure and efficient clip closure 2 . These effects are consistent with the reported advantages of the EndoFaster system 3 4 . The procedure was completed without intraprocedural or early adverse events, and the patient recovered uneventfully. This case suggests that robot-assisted closure can be a safe and effective option for managing post-ESD defects, particularly in anatomically challenging locations. Further prospective clinical studies are warranted to validate its broader applicability.

Endoscopy_UCTN_Code_TTT_1AO_2AO
